# A comparison of safety and efficacy between long-term DAPT and intensive statins combined with short-term DAPT for acute ischemic stroke

**DOI:** 10.1186/s40001-023-01115-5

**Published:** 2023-04-21

**Authors:** Ting Deng, Xiaomeng Liu, Wei He, Jingmian Chen, Xiaohua Yao, Lushan Liu, Tong Zhang, Haitao Lu

**Affiliations:** 1 Emergency Department of Beijing Bo’ai Hospital, School of Rehabilitation Medicine, Capital Medical University, Beijing, 100068 China; 2 Neurology Department of Beijing Bo’ai Hospital, School of Rehabilitation Medicine, Capital Medical University, Beijing, 100068 China

**Keywords:** Acute ischemic stroke, Double antiplatelet therapy, Intensive statin therapy, Safety and efficacy

## Abstract

**Objectives:**

The current study compared the safety and efficacy of long-term dual antiplatelet therapy (DAPT, aspirin plus clopidogrel) and intensive rosuvastatin with short-term DAPT for acute ischemic stroke (AIS).

**Methods:**

A total of 220 patients were enrolled 72 h after the onset of mild to moderate AIS, and divided into a control group treated with 21-day DAPT and a study group treated with intensive rosuvastatin with 7-day DAPT on a voluntary basis. The primary outcome was recurrent ischemic stroke and hemorrhage during a 90-day follow-up period in an intention-to-treat analysis. The secondary outcome was clinical efficacy with respect to alleviating existing focal nerve defect symptoms. A Cox proportional-hazards model was used to evaluate treatment differences.

**Results:**

Clinical efficacy was evident in 87.3% of patients in the study group, compared with 84.3% in the control group (*p = 0.042*). Recurrent ischemic stroke occurred in 9 patients (7.6%) in the study group and in 9 (8.8%) in the control group (*p* = 0.767). Hemorrhage occurred in 6 patients (5.1%) in the study group and in 15 (14.7%) in the control group (*p* = 0.023). In comparisons of levels of ALT, AST, LDH, and CK in the two groups before and 2 weeks after therapy, only CK differed significantly (*p* < 0.001).

**Conclusions:**

Compared to long-term DAPT, intensive rosuvastatin with short-term DAPT was equivalent in reducing the risk of recurrent ischemic stroke. It alleviated symptoms more rapidly, and significantly reduced the risk of bleeding, without causing an increase in transaminase or muscle enzymes.

***Clinical trial registration*:**

China Clinical Trial Registration Center (ChiCTR1800017809)

## Highlights

- Studies have shown that 21-day dual antiplatelet therapy (DAPT) with aspirin and clopidogrel reduced the risk of recurrent cerebral infarction in minor acute ischemic stroke (AIS) patients at the first 90 days, but also increased the risk of bleeding, especially after the 7th day of DAPT.
- The results of this manuscript entitled "A comparison of safety and efficacy between long-term DAPT and intensive statins combined with short-term DAPT for acute ischemic stroke" showed that compared with 21-day DAPT, intensive rosuvastatin plus 7-day DAPT significantly reduced the risk of bleeding without increasing recurrent cerebral infarction in mild to moderate AIS patients.

## Background

Stroke is still the leading cause of death in China [1], and its incidence has increased year by year [2]. The age standardized incidence rate of ischemic stroke in China increased by 34.7% from 1990 to 2019, and it accounted for 82.6% of strokes [3, 4]. The annual recurrence rate was approximately 18.0% [5], causing huge psychological burdens on patients, and economic burdens on families and society [6]. There is, therefore, a need to reduce the incidence and recurrence rate of ischemic stroke, and alleviate resulting nerve defect symptoms as soon as possible. Intensive statin therapy is the cornerstone of acute coronary syndrome [7, 8] and can reduce the risk of recurrent ischemic stroke [9, 10]. Abnormal platelet aggregation is the main pathogenesis of acute ischemic stroke (AIS) [11]. Double antiplatelet therapy (DAPT) consisting of aspirin and clopidogrel can significantly reduce recurrent ischemic stroke in the first 90 days in patients with acute minor ischemic stroke or high-risk transient ischemic attack (TIA) [12–15], but also increases the incidence of bleeding events [13, 16]. To identify a more appropriate DAPT regimen for AIS, the current study compared the efficacy and safety of intensive rosuvastatin with short-term (7 days) DAPT (aspirin + clopidogrel) and long-term (21 days) DAPT in patients with mild to moderate AIS.

## Materials and methods

### Patients

Patients with mild to moderate AIS admitted to the Emergency Department between January 2017 and December 2019 were recruited. The study was reviewed and approved by the Medical Ethics Committee of our hospital (approval number 2016-065-1). The patients and their legal proxies were informed of the study and signed a consent form.

The inclusion criteria were (1) age ≥ 18 years; (2) onset to medication time (OMT) ≤ 72 h; (3) National Institutes of Health Stroke Scale (NIHSS) scores at registration ≤ 7 points; and (4) head computed tomography (CT) at registration excluding intracranial hemorrhage, and brain magnetic resonance imaging (MRI) showing new infarction lesions within 3 days after registration. The exclusion criteria were (1) intravenous thrombolysis/arterial thrombectomy; (2) taking anticoagulants; (3) other brain diseases such as intracranial tumors, vascular malformations, abscesses, encephalitis, and TIA; (4) severe function disorders of the heart, liver, kidney, lung, immune system, or coagulation, or malignant tumors; and (5) pregnancy or women preparing for pregnancy within 3 months.

**Fig. 1:**
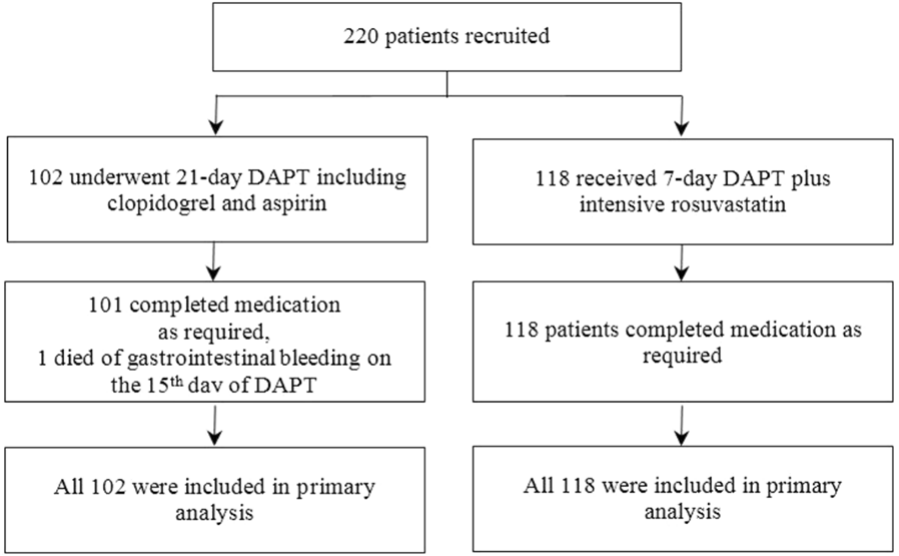
Enrollment and outcomes (intention-to-treat analysis).

In total, 220 patients with focal nerve defect symptoms (excluding simple dizziness/vertigo, ataxia, and sensory or visual impairment) were confirmed via brain MRI, including 102 in the control group and 118 in the study group. There were no withdrawals or unrelated deaths except for 1 patient who died of massive gastrointestinal bleeding in the control group. She was an 84-year-old woman with a known atrial fibrillation and enrolled 70 h after the onset of AIS. During the treatment her existing nerve defect symptoms worsened, she suffered gastrointestinal bleeding on the 12th day, and she died of massive gastrointestinal bleeding on the 15th day of DAPT [17]. Because this patient did not affect efficacy evaluations at 21 days or 90 days, she was not excluded from the statistical analysis (Fig. 1).

NIHSS is the most commonly used scale for assessing stroke severity, and scores range from 0 to 43. The higher the score the greater the defect, and 0–4 is considered mild, 5–15 is considered moderate, and > 15 is considered severe [18]. Because most severe AIS patients require intravascular treatment (thrombolysis/thrombectomy), only patients with the baseline score of NIHSS (bNIHSS)≤ 7 points were included in the study. bNIHSS ≤ 4 points and bNIHSS > 4 points (5–7 points) were used as the basis for stratification.

### Therapy regimen

Due to polymorphism of the liver cytochrome P-450 isoenzyme (clopidogrel-activating enzyme) gene in Asian populations, the clinical efficacy of clopidogrel remains uncertain [19]. Therefore, the DAPT regimen in the current study included aspirin as the main body and clopidogrel as the synergy. The control group received aspirin 100 mg/day (Bayer, 100 mg per tablet, initial dose 300 mg) for 90 days, clopidogrel 75 mg/day (Sanofi, 75 mg per tablet, initial dose 75–300 mg based on clinical symptoms) for 21 days, and rosuvastatin 10 mg/day (Nanjing Chia Tai-Tianqing Pharmaceutical Co., Ltd., 10 mg per tablet) for 90 days.

The study group received aspirin 100 mg/day (100 mg per tablet, initial dose 300 mg) for 90 days, clopidogrel 75 mg/day (75 mg per tablet, initial dose 75–300 mg based on clinical symptoms) for 7 days, and rosuvastatin 20 mg/day (10 mg per tablet) for 21 days, then 10 mg/day for 69 days. The basic treatments were the same and the follow-up time was 90 days in both groups.

### Measurement of biomarkers

Venous blood was collected from all patients at registration (before treatment) and after 2 weeks of treatment (14 ± 3 days), and the levels of alanine aminotransferase (ALT), aspartate aminotransferase (AST), lactate dehydrogenase (LDH), and creatine kinase (CK) were measured using an automatic BS-800M biochemical analyzer (Mindray, China).

### Criteria used to evaluate clinical efficacy

#### Clinical efficacy over a 21-day period

Yoo et al. [20] reported that a change of  ≥ 2 NIHSS points indicates that a patient’s nerve defect symptoms have changed significantly (improvement or deterioration), and this threshold was used as the basis for judging therapeutic efficacy in the present study. NIHSS scores were determined at eight different time points; before therapy (bNIHSS), 2, 12, 24, and 48 h after therapy, and 1, 2, and 3 weeks after therapy. When the score decreased by ≥ 2 points, one therapeutic effectiveness event and the time of the effectiveness event were recorded (each case was only recorded once). Patients with bNIHSS = 1 and those with an increase in NIHSS score within a 21-day period were treated as deletion events. All patients’ NIHSS scores were jointly assessed by two emergency physicians who had received formal training.

#### Recurrent ischemic stroke events within 90 days

These events were defined as the appearance of a new focal nerve defect symptom within the 90-day follow-up period, or the rapid worsening of existing focal nerve defect symptoms with a duration of  ≥  24 h, as determined by brain MRI. Non-ischemic factors were excluded (i.e*.*, intracranial infection, trauma, tumor, epilepsy, serious metabolic diseases, and degenerative neurological diseases). When a recurrent ischemic event occurred, one failure event (recurrent ischemic stroke) and the failure time were recorded.

#### Bleeding events within 90 days

All bleeding events in both groups were recorded over a 90-day period after initial medication. There were several bleeding sites, including intracranial, gastrointestinal, the skin, and the mucosa. All patients with bleeding, including intracranial hemorrhage confirmed by cranial CT, along with gastrointestinal bleeding with occult blood positive for vomit and stools, were recorded as one failure (bleeding) event along with the failure time. In accordance with the Global Utilization of Streptokinase and Tissue Plasminogen Activator for Occluded Coronary Arteries criteria, bleeding was classified as mild, moderate, or severe, where severe bleeding referred to hemorrhage resulting in hemodynamic compromise requiring vasoactive drug support, fluid or blood replacement, or surgical intervention [17].

### Statistical analysis

The study used an incomplete randomized controlled trial design, with type I error *α* = 0.05 and a test power of (*1 − β*) = 0.95. G*Power software (Dusseldorf University, Germany) was used to estimate the required sample size. The moderate intensity effect size recommended by the software was 0.3, and the total sample size indicated was 220, with a control group to study group ratio of approximately 1:1. A total of 220 patients were recruited, including 102 in the control group and 118 in the study group.

SPSS 25.0 software (IBM Corporation, Armonk, NY, USA) was used for data analysis. Because baseline characteristics data in the two groups did not conform to a normal distribution, measurement data are expressed as median and interquartile range and were analyzed via the Mann–Whitney *U* test, and frequency data are expressed as percentages and were analyzed via the chi square test. The log-rank survival function model was used to compare clinical efficacy in the two groups within a 21-day period. A Cox proportional hazards model was used to evaluate recurrent ischemic stroke and hemorrhage within 90 days in the two groups. Age, gender, OMT, systolic blood pressure at registration, bNIHSS, and other factors were stratified to analyze associations with recurrent ischemic stroke in the two groups. *P* < 0.05 was considered statistically significant.

## Results

### Baseline patient characteristics

A total of 220 patients with mild to moderate AIS were enrolled, their median age was 65.5 years, and 24.1% were women. There was a history of hypertension in 83.6% of patients, 42.2% had diabetes, 17.3% had hyperlipidemia, 31.4% had prior ischemic stroke, and 32.7% were current or former recipients of antiplatelet therapy. There were no significant differences in baseline characteristics between the two groups (all *p* > 0.05) (Table 1).

**Table 1:** Baseline characteristics of the patients

Characteristic	All cases (*n* = 220)	Control group (*n* = 102)	Study group (*n* = 118)	*Z*/*χ*^2^	*P*-Value
Age (IQR)/year	65.50 (58.00, 76.00)	65.00 (57.75–76.00)	66.00 (58.00–76.00)	−0.082	0.935
Female (*n*, %)	53 (24.09)	25 (24.51)	28 (23.73)	0.018	0.893
SBP (IQR)/mmHg	154.00 (140.00, 172.0)	155.50 (138.00–176.00)	154.00 (142.75–170.50)	−0.398	0.690
DBP (IQR)/mmHg	90.00 (80.00, 103.00)	89.00 (77.50–103.00)	90.00 (81.00–103.00)	−1.113	0.266
OMT (IQR)/*h*	16.00 (5.00, 36.00)	13.00 (4.75–27.00)	18.00 (5.00–48.00)	−0.761	0.447
bNIHSS (IQR)	4.00 (3.00, 5.00)	4.00 (3.00–5.00)	4.00 (3.00–4.25)	−1.223	0.221
Medical history (*n*, %)					
Hypertension	184 (83.64)	85 (83.33)	99 (83.90)	0.013	0.910
Diabetes	93 (42.27)	45 (44.12)	48 (40.68)	0.265	0.607
Hyperlipidemia	38 (17.27)	19 (18.63)	19 (16.10)	0.244	0.621
Atrial fibrillation	31 (14.09)	16 (15.69)	15 (12.71)	0.400	0.527
Prior ischemic stroke	69 (31.36)	32 (31.37)	37 (31.36)	0.000	0.998
Prior antiplatelet	72 (32.73)	37 (36.27)	35 (29.66)	1.087	0.297
Median baseline of various enzymology before medication (IQR) – U/L					
ALT	16.80 (12.20, 23.40)	17.55 (11.95–25.08)	16.45 (12.73–22.30)	−0.238	0.812
AST	16.60 (12.80, 21.65)	15.95 (11.78–20.93)	17.15 (13.55–22.00)	−1.494	0.135
LDH	171.00 (149.00, 200.00)	173.50 (150.00–200.00)	170.00 (147.50–200.25)	−0.713	0.476
CK	74.50 (54.25, 105.50)	75.50 (58.75–113.25)	74.50 (52.00–101.25)	−0.825	0.409
Median of various enzymology in 2 weeks (14 ± 3 d) after medication (IQR) – U/L					
ALT	17.35 (12.03, 27.18)	18.15 (12.00–27.70)	16.85 (12.00–25.68)	−0.832	0.406
AST	16.40 (13.20, 21.78)	16.10 (13.00–21.25)	16.85 (13.28–22.03)	−0.658	0.510
LDH	168.00 (151.00, 187.00)	168.00 (152.75–187.00)	168.00 (147.50–188.25)	−0.219	0.827
CK	71.00 (46.25, 100.00)	66.00 (46.00–95.25)	72.00 (49.00–102.25)	−1.165	0.244

There were no significant differences between study group and control group for any characteristic

*SBP* systolic blood pressure, *DBP* diastolic blood pressure, *OMT* onset to medication time, *bNIHSS* the baseline score of National Institutes of Health Stroke Scale, *ALT* alanine aminotransferase, *AST* aspartate aminotransferase, *LDH* lactate dehydrogenase, *CK* creatine kinase

### Clinical efficacy in the two groups

**Fig. 2:**
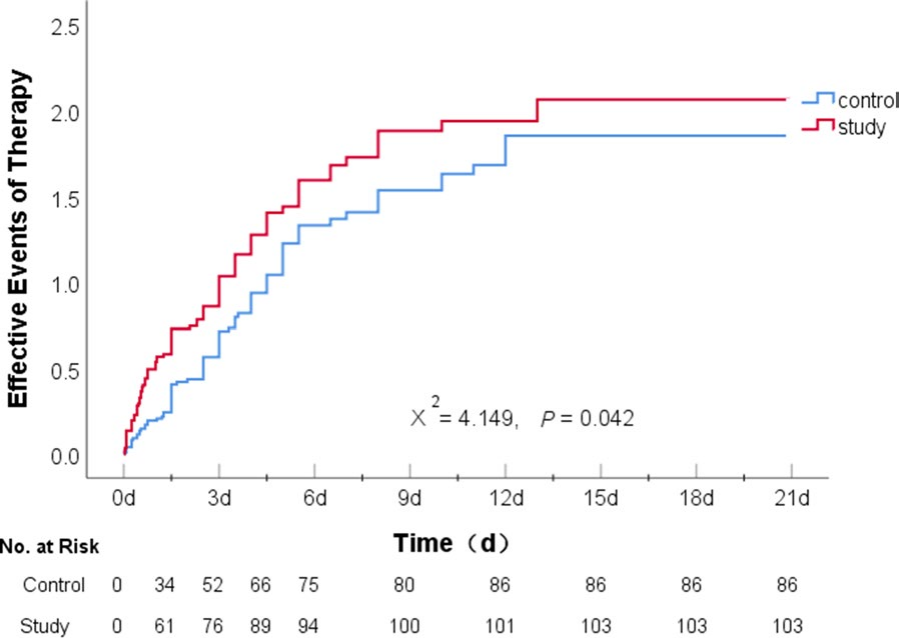
Probability of therapeutic efficacy events at 21 days

Within a 21-day period, there were 86 (84.31%) therapeutic effectiveness events in patients in the control group and 103 (87.29%) in patients in the study group. A log-rank survival function model revealed a significant difference between the two groups (χ^2^ = 4.149, *p* = 0.042). The effectiveness events mainly occurred within the 1 week after initial medication (75 patients in the control group and 94 in the study group) and were more prevalent in the study group within 1.5 days (34 patients in the control group and 61 in the study group), suggesting that intensive statins combined with short-term (7-day) DAPT was more effective in terms of relieving existing nerve defect symptoms in patients with mild to moderate AIS (Table 2 and Fig. 2).

**Table 2:** Therapeutic efficacy within a 21-day period*

Outcomes	Effective—no (%)		Log Rank (Mantel-Cox)	
	Cases with events	Incidence of events (%)	Chi-Square	*p* value
Control group (*n* = 102)	86	84.3	4.149	0.042
Study group (*n* = 118)	103	87.3		

Therapeutic efficacy events were recorded when NIHSS score decreased by ≥ 2 points, in accordance with Yoo et al. [20]

### Recurrent ischemic stroke in the two groups

**Fig. 3:**
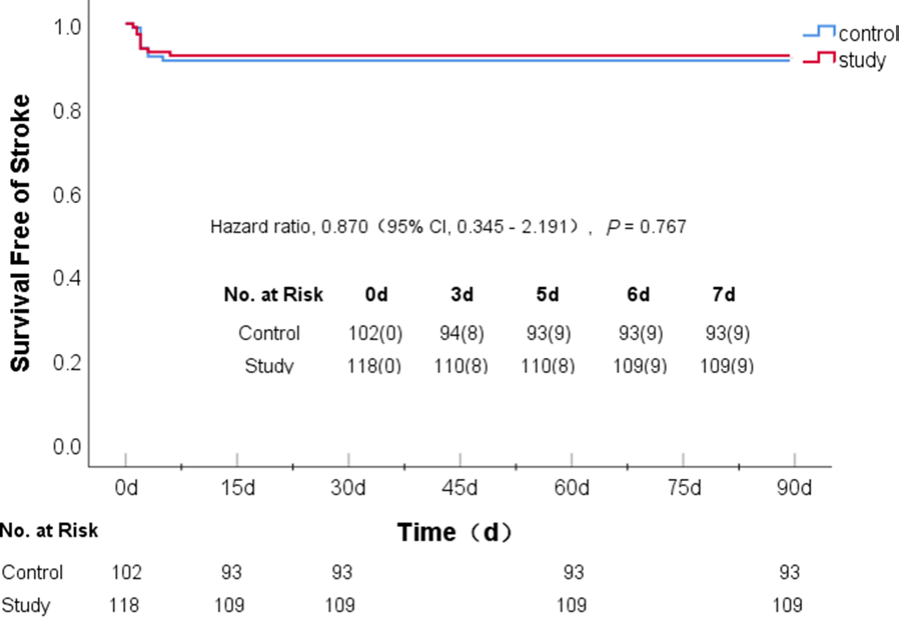
Probability of survival free of recurrent ischemic stroke within 90 days

Within a 90-day follow-up period there were 9 (8.8%) recurrent ischemic stroke events in the control group and 9 (7.6%) in the study group. In a Cox proportional hazards model there was no significant difference between the two groups (*p* = 0.767), although the risk of recurrent ischemic stroke was reduced by 13% in the study group compared to the control group (hazard ratio [HR] 0.870; 95% confidence interval [CI] 0.345–2.191). The recurrent ischemic stroke events in the two groups mainly occurred within 3 days of initial medication (8 cases in the control group and 8 cases in the study group; Table 3 and Fig. 3). This suggested that the two regimens of the intensive statins with short-term DAPT and long-term DAPT had equivalent effects on reducing the risk of recurrent stroke in AIS patients at 90 days. Intensive statin therapy with short-term DAPT was slightly better than the long-term DAPT regimen.

**Table 3:** Efficacy and safety outcomes

Outcomes	Control group		Study group		Hazard ratio (95% CI)	*p*-value
	Cases with events	Incidence of events (%)	Cases with events	Incidence of events (%)		
Stroke	9	8.8	9	7.6	0.870 (0.35–2.19)	0.767
Bleeding	15	14.7	6	5.1	3.008 (1.17–7.75)	0.023
Severe^a^	1	1.0	0	0.0		
Mild	14	13.9	6	5.1	3.039 (1.18–7.84)	0.021

^a^One patient in the control group died of massive gastrointestinal bleeding

**Fig. 4:**
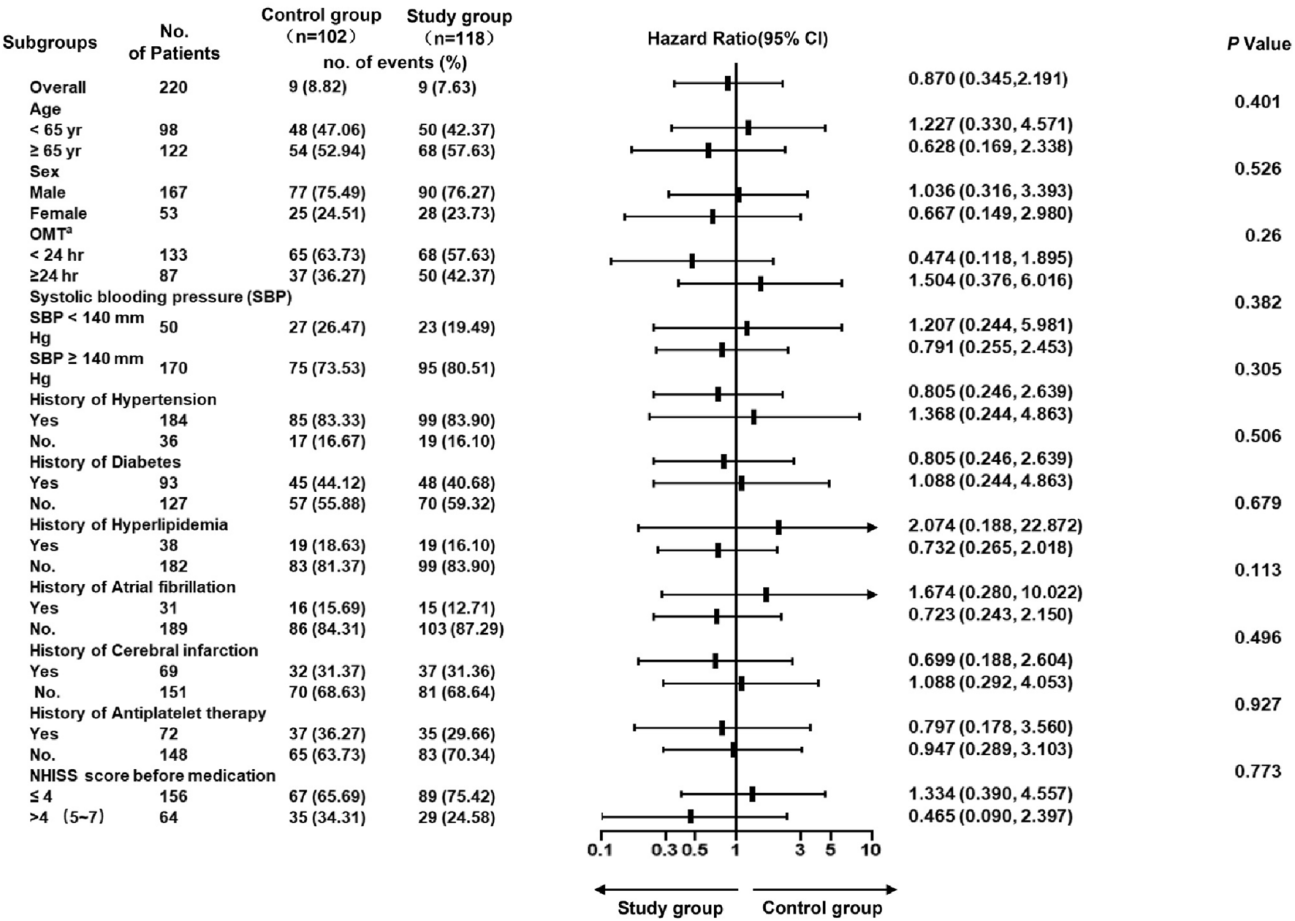
Hazard ratio for recurrent ischemic stroke in subgroups within 90 days.

To ensure the accuracy of evaluation of the risk of recurrent ischemic stroke, the baseline characteristics of patients in the two groups were analyzed with respect to interaction between subgroups and their respective therapy regimen. According to the DAWN trial [21] and Epidemiological data in China, the OMT cut-off value is 24 h (< 24 h and ≥ 24 h), and the age cut-off value is 65 years (< 65 years and ≥ 65 years) [22]. There was no significant interaction between therapy regimen and any subgroup (all *p* > 0.20), indicating that the two regimens had the same effects on reducing the risk of recurrent cerebral infarction (Fig. 4).

### Bleeding events in the two groups

**Fig. 5:**
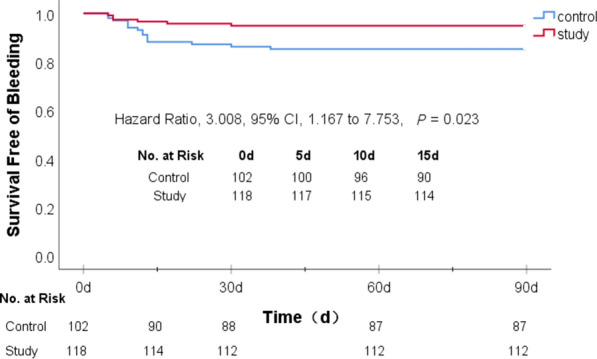
Probability of survival free of bleeding within 90 days.

With respect to patients with bleeding events within 90 days, there were 15 (14.7%) in the control group including the patient with massive gastrointestinal bleeding, and there were 6 (5.1%) in the study group. In a Cox proportional hazards model there was a significant difference between the two groups (*p* = 0.023). The risk of bleeding in the control group was threefold higher than that in the study group (HR 3.008; 95% CI 1.167–7.753). Bleeding events mainly occurred from the 5th to the 15th day after initial medication (12 patients in the control group and 4 in the study group) (Table 3 and Fig. 5), suggesting that DAPT was the main cause of bleeding events. The risk of bleeding in the long-term DAPT group was significantly higher than that in the intensive statins plus short-term DAPT group.

### Levels of transaminase and muscle enzymes before and 2 weeks after initial medication

No patients exhibited a threefold increase in levels of transaminase or muscle enzymes in either group within 21 days after the initiation of medication. There were no significant differences in levels of ALT, AST, or LDH before and 2 weeks (14 ± 3 days) after treatment in either group (*p* > 0.05). In the control group, CK levels after medication initiation were significantly lower than pre-medication levels (*p* = 0.001) (Table 4). These results showed that the two regimens did not increase levels of transaminase or muscle enzymes.

**Table 4:** Comparison of levels of ALT, AST, LDH, and CK before and 2 weeks after therapy

		Control group				Study group			
		ALT	AST	LDH	CK	ALT	AST	LDH	CK
Before	25%	12.18	10.58	152.00	54.00	12.75	13.55	147.50	52.00
	Median	18.55	14.80	171.00	69.00	16.45	17.15	170.00	74.50
	75%	26.38	20.68	197.75	104.00	22.30	22.00	200.25	101.25
After	25%	12.00	13.00	152.75	46.00	12.00	13.28	147.50	49.00
	Median	18.15	16.10	168.00	66.00	16.85	16.85	168.00	72.00
	75%	27.70	21.25	187.00	95.25	25.68	22.03	188.25	102.00
*P* Value(2-tailed)		0.217	0.520	0.292	0.000	0.498	0.357	0.973	0.601

*ALT* alanine aminotransferase, *AST* aspartate aminotransferase, *LDH* lactate dehydrogenase, *CK* creatine kinase

## Discussion

The current study compared the safety and efficacy of a 21-day DAPT regimen and intensive rosuvastatin plus a 7-day DAPT regimen in patients with mild to moderate AIS. The two regimens had the same effectiveness with respect to reducing the risk of recurrent ischemic stroke within 90 days. Compared to the 21-day DAPT regimen, the regimen of intensive rosuvastatin plus 7-day DAPT alleviated existing focal nerve defect symptoms more effectively, and significantly reduced the risk of bleeding without causing an increase in transaminase or muscle enzymes.

The equivalence of the two regimens in reducing the risk of recurrent ischemic stroke within 90 days suggests that an intensive statins plus short-term (7-day) DAPT regimen could replace long-term (21-day) DAPT regimens in patients with mild to moderate AIS. It is possible that there is a material basis for the role of intensive statins in the early stages of AIS. Non-cardiogenic AIS is a form of atherosclerotic cardiovascular disease (ASCVD) [23]. Increased levels of low-density lipoprotein cholesterol (LDL-C) and non-high-density lipoprotein cholesterol (non-HDL-C) in circulation are the root cause of arterial atherosclerosis and central to the occurrence of ASCVD events [24, 25]. Intensive statin therapy can effectively reduce the levels of LDL-C and non-HDL-C in circulation, inhibit the formation of thrombus, and promote thrombolysis and plaque stability [26, 27], thereby reducing the occurrence of acute events related to ASCVD [28, 29] and significantly reducing the risk of recurrent ischemic stroke [9, 10]. Moderate intensity statin therapy can significantly reduce the risk of cardiovascular and cerebrovascular diseases by reducing LDL-C levels, especially in elderly patients [30]. For every 1 mmol/L decrease in LDL-C, the risk of major cardiovascular events decreases by 15% in people aged under 75 years, and by 26% in those aged over 75 years [31]. Compared to conventional doses, intensive statins can significantly reduce the risk of recurrent stroke within 90 days [32].

The intensive statins plus short-term (7-day) DAPT regimen administered in the current study could effectively alleviate clinical symptoms. This is because DAPT including aspirin and clopidogrel effectively inhibited platelet aggregation and reduced thrombosis, and the multiple effects of intensive statin therapy also played a key role. Studies have shown that statins exhibit strong anti-inflammatory and antioxidant functions [33] and can improve atherosclerosis, reduce vascular plaques, and prevent the formation of thrombosis [26, 27]. Statins can increase nitric oxide levels, expand cerebral vessels, increase cerebral blood flow, and reduce the area of infarction by upregulating levels of endothelial nitric oxide synthase, thereby relieving nerve defect symptoms [34, 35]. Moreover, these effects are dose dependent [26, 29].

AIS is associated with multiple factors, including hypertension, diabetes, hyperlipidemia, smoking, and drinking alcohol. Most patients usually have accompanying gastric mucosal erosion or gastroduodenal ulcers, and cannot tolerate long-term high-dose DAPT. Previous studies have shown that bleeding events mainly occur after the 7th day of DAPT [13], which is consistent with our findings indicating that DAPT was a major factor in bleeding events. The intensive rosuvastatin combined with a 7-day DAPT regimen exhibited clinical efficacy while effectively reducing the risk of bleeding.

The intensive rosuvastatin plus short-term DAPT regimen in the study group did not result in adverse events such as significant increases in transaminase or muscle enzyme levels. This is because rosuvastatin is a water-soluble statin that can only enter liver cells via specific carrier proteins located in the liver cell membrane, to inhibit cholesterol synthesis. Rosuvastatin rarely leads to serious side effects such as rhabdomyolysis [36, 37], and rarely inhibits cholesterol synthesis in the adrenal glands, heart, brain, or other tissues [36].

The results of the current study are objective, reliable, and highly replicable in clinical practice. However, due to the single center, small sample size, incompletely randomized and controlled design of the study, and the lack of hierarchical analysis based on different stroke types, it is inevitable that there are some deviations that need to be addressed in future studies with larger sample sizes.

## Conclusion

Compared to a long-term (21-day) DAPT regimen, an intensive rosuvastatin with short-term (7-day) DAPT regimen was equally effective for reducing the risk of recurrent ischemic stroke within 90 days and alleviated existing focal nerve defect symptoms more rapidly, and it significantly reduced the risk of bleeding without increasing levels of transaminase or muscle enzymes.

## Data Availability

The data used to support the findings of this study are available from the corresponding author upon requests.

## Abbreviations

AIS: Acute ischemic stroke
ALT: Alanine aminotransferase
ASCVD: Atherosclerotic cardiovascular disease
AST: Aspartate aminotransferase
bNIHSS: The baseline score of NIHSS
CK: Creatine kinase
CT: Computed tomography
DAPT: Dual antiplatelet therapy
DBP: Diastolic blood pressure
LDH: Lactate dehydrogenase
LDL-C: Low-density lipoprotein cholesterol
MRI: Magnetic resonance imaging
NIHSS: National Institutes of Health Stroke Scale
OMT: Onset to medication time
TIA: Transient ischemic attack
